# Moving Target Tracking through Distributed Clustering in Directional Sensor Networks

**DOI:** 10.3390/s141224381

**Published:** 2014-12-18

**Authors:** Asma Enayet, Md. Abdur Razzaque, Mohammad Mehedi Hassan, Ahmad Almogren, Atif Alamri

**Affiliations:** 1 Green Networking Research (GNR) Group, Deptartment of Computer Science and Engineering, Facutly of Engineering and Technology, University of Dhaka, Dhaka 1000, Bangladesh; E-Mail: asmaenayet@gmail.com; 2 College of Computer and Information Sciences, Chair of Pervasive and Mobile Computing, King Saud University, Riyadh 11543, Saudi Arabia; E-Mails: mmhassan@ksu.edu.sa (M.M.H.); ahalmogren@ksu.edu.sa (A.A.); atiffksu.edu.sa (A.A.)

**Keywords:** target tracking, area coverage, energy, cluster

## Abstract

The problem of moving target tracking in directional sensor networks (DSNs) introduces new research challenges, including optimal selection of sensing and communication sectors of the directional sensor nodes, determination of the precise location of the target and an energy-efficient data collection mechanism. Existing solutions allow individual sensor nodes to detect the target's location through collaboration among neighboring nodes, where most of the sensors are activated and communicate with the sink. Therefore, they incur much overhead, loss of energy and reduced target tracking accuracy. In this paper, we have proposed a clustering algorithm, where distributed cluster heads coordinate their member nodes in optimizing the active sensing and communication directions of the nodes, precisely determining the target location by aggregating reported sensing data from multiple nodes and transferring the resultant location information to the sink. Thus, the proposed target tracking mechanism minimizes the sensing redundancy and maximizes the number of sleeping nodes in the network. We have also investigated the dynamic approach of activating sleeping nodes on-demand so that the moving target tracking accuracy can be enhanced while maximizing the network lifetime. We have carried out our extensive simulations in ns-3, and the results show that the proposed mechanism achieves higher performance compared to the state-of-the-art works.

## 1. Introduction

Wireless sensors are miniature devices integrated with data processing, physical sensing and communication units, which provide great research interest for a wide range of applications [1-5]. Sensors can be categorized as omnidirectional and directional sensors. An omnidirectional sensor can equally detect the surrounding environment in any direction with its omnidirectional antenna. Unlike omnidirectional sensors, a directional sensor has a limited range of sensing and communication capabilities, since it can detect only a certain field of vision or a limited direction. A good number of practical directional sensor motes are now available in the market, including cameras, infrared and ultrasonic sensors [6,7]. In a directional sensor network (DSN), the communication area of a sensor is a sector rather than a disk. Directional sensors improve the quality of sensing and scale down the interference and fading, which, in turn, enhance the network performance, as well as the lifetime [8].

Moving target tracking is an important application that requires sensing nodes to cooperate with each other in order to achieve a good outcome [5,9,10]. Accurate path detection, low-cost data reporting, maximum performance without losing data packets and maximizing the network's lifetime have always been the critical goals for moving target tracking in wireless sensor networks. The problem has been well studied in omni-directional sensor networks [11-18]. These solutions are not applicable for DSNs, as the directionality of sensor devices imposes new research challenges in the domain. Even simple modified versions of these approaches are not applicable in DSNs. In the literature, a few research works are found for moving target tracking in directional sensor networks [8,19], and we have a further scope for research.

Hu *et al.* [20] addressed the location estimation problem of a moving target using a team of mobile robots in which directional sensors are integrated. In [19], highly directional sensors are used to detect the motion of the target, whose field of vision is a line. To overcome the highly convex optimization problem, an adaptive basis algorithm (ABA) is introduced. The ABA estimates the trajectory, direction and field of the objects and sensing lines. Zijan *et al.* [8] used co-operative DSN, where each sensor gives approximate direction information about the moving target to the sink in real time in a distributed manner. The authors proposed a sector-based sensor, where each node identifies the target's presence or absence and also calculates the location of the target based on target detection information from neighboring sensor nodes. These two approaches increase the computation overhead, redundant sensing information to the sink and erroneous information reception from all of the nodes. Therefore, the connectivity of the sink to each of the nodes in the sensor network is an energy hungry and overloaded process. In addition, poor coordination among sensing nodes and the excessive transfer of messages to the sink might reduce the tracking accuracy.

In this paper, we introduce a distributed clustering approach to solve the moving target tracking problem, where each cluster head coordinates the computation and communication of target sensing data with the sink. This work is motivated by the fact that the minimum number of sensor devices, required for accurate target tracking, will remain active under each cluster head, so that the network lifetime is maximized. Each cluster head (CH) determines the active sensing member nodes and their sensing directions in order to cover its working sector area. The sensor nodes transmit target detection information to their CH, which estimates the location of the target more precisely by exploiting sensing data from multiple member sensing nodes. The CH then sends the target location information to the sink. To the best of our knowledge, there has been no work in the literature that exploits CHs to solve the moving target tracking problem in an energy-efficient way. Our approach is fully distributed, and it exploits single-hop neighborhood information only. The main contributions of this paper are summarized below.

- The novelty of this work lies in the development of a cluster-based solution to the moving target tracking problem in directional sensor networks (DSNs).
- The cluster head-based optimization of the number of active nodes within a cluster minimizes the sensing redundancy and maximizes the number of sleeping nodes in the network.
- The cluster head-based location estimation and data processing reduces the network contention and computational overhead of energy-constrained sensor devices and ensures accuracy.
- The moving target tracking through distributed clustering (MTDC) system is capable of tracking a target originating from anywhere in the monitored area, as well as entering into the terrain from outside.
- The results of performance evaluations, carried out in ns-3 [21], show that our proposed MTDC system achieves better performances compared to state-of-the-art mechanisms in terms of tracking accuracy, active sensor nodes, standard deviation of residual energy and network lifetime.

The remainder of the paper is organized as follows. Section 2 contains a study on the related works in this field of research. The network model and assumptions are presented in Section 3, and Section 4 gives explicit insight into our proposed clustering, gateway selection, active node selection and tracking algorithms for directional sensor networks. Section 5 presents the performance evaluation of the algorithm. Finally, we conclude the paper in Section 6.

## 2. Related Works

The coverage problem for fixed targets has been addressed in [22,23] using directional sensor nodes, where the number of sensors required to be deployed is minimized either in a centralized or in a distributed way. None of them investigate the problem of moving target tracking or enhancement of target tracking accuracy. Being an enriched area of research, moving target tracking, in energy-efficient way, in wireless sensor networks (WSN) has received many proposals over the last few years [8,12,14,19]. The existing mechanisms can be classified into several categories: target tracking in an omnidirectional sensor network and target tracking in a directional sensor network are the significant types. The principle research area related to our work is target tracking using distributed clustering in a directional sensor network.

A number of target tracking mechanisms have been proposed in WSN. The algorithms presented in [24,25] have the central node, which gathers the target's binary information from all of the sensors in the network and applies a particle filter on that information to update the target's track. However, transmitting information of one single node from each sensor is energy hungry; yet, this centralized approach is not reliable, and also, the particle filters are expensive to compute.

Kim *et al.* [12] improved a distance-based weight calculation for each sensor that detects the target. Here, the authors developed a linear approximation model for target's trajectory by allocating a small flexible window to the previous measurements, and a straight line segment is used to represent the target's trajectory in that window. This algorithm computes the weighted average of the sensors, which detected the target and is used as an estimated point on the path of the target. Each estimated point is determined from the recent path and estimated the target's velocity, and the line equation helps to determine the target's location. However, this method needs time synchronization across the network, as well as complexity while calculating. Hence, the tracking is not in real time, but delayed.

All of the previous research used omnidirectional sensor networks, which can estimate the target's location roughly, but cannot get the direction because of the wide field of view. Plarre *et al.* [19] treated the problem of tracking objects using highly directional sensors whose field of vision is apparently a straight line. Here, a sensor detects an object when it crosses the line and keeps a record of the time of detection. From the time information, a sensor estimates the trajectory using an *ad hoc* coordinate system. However, this approach uses a highly directional sensor, and the field of vision is much less, which cannot cover the entire area. Wang *et al.* [8] proposed a distributed target tracking algorithm (RDTT) in a directional sensor network in which each sensor is divided into sectors and can detect the target's presence or absence in the sectors. Here, each sensor estimates and calculates the target's location using the coordination among the neighboring sensors. However, here, each sensor communicates with the neighboring sensors, and the sink increases the network traffic and data loss, which will lead to reduced accuracy in target tracking and energy wastage.

In this paper, we develop a distributed cluster-based solution to the problem of moving target tracking with improved accuracy and reliability in directional sensor networks. The existing clustering algorithms, SPAN [26] and FLOC [27], and data delivery framework, Sprinkler [28], for omnidirectional sensor networks are not usable in our MTDC system. We develop a basic clustering algorithm (A similar algorithm has been presented in one of our recent conference papers [29]) for DSNs and use the cluster heads (CHs) to execute the target tracking algorithms. To the best of our knowledge, cluster-based target tracking in a directional sensor network has not been introduced in any of the existing works. Our concept has much similarity with [8], except the following distinct differences. First, we introduce a distributed clustering approach at the network deployment stage to address the communication overhead. Here, a cluster head is selected in a distributed manner, and a cluster head selects gateway nodes for the communication to the sink. The cluster head calculates the location of the target and communicates to the sink. Hence, the network traffic and energy wastage is reduced. Second, in order to increase the network's lifetime and to save energy, we develop an active node selection algorithm that runs in each cluster head to cover an entire cluster area with the minimum number of active sensor nodes initially. Only when a target's presence is detected, the cluster head wakes up the neighboring sleeping nodes. Finally, the location calculation and communication to the sink is the cluster head's (CH) responsibility in our protocol. A CH does this by gathering the target detection information from the cluster members.

## 3. Network Model

We assume a directional sensor network (DSN) is comprised of 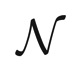 stationary sensor nodes placed over a finite two-dimensional planar region. The sensors are deployed randomly with uniform distribution and high density, so that the coverage and connectivity is maintained [30]. The nodes form a cluster based data communication network, so that the cluster heads can communicate data to the sink node in a multi-hop fashion. We assume that the sensors reliably detect the presence of targets, *i.e.*, the location of a target is reported if it is within the sensing range of a sensor. The sensors send the sensed data to the cluster head, which determines the target location and communicates with the sink. At the network deployment stage, each node is denned by three tuples < *ID*, (*x*,*y*),*E_init_* >, where *ID* is the unique identifier of a node, (*x*,*y*) is the Cartesian location of a sensor (determined by GPS or any other localization method [31,32]) and *E_init_* is its residual energy. Each node knows the above three tuples'; information of each of its neighbor nodes by a neighbor discovery protocol [33]. We assume that the sensing and communication ranges of the sensor devices are identical and the nodes have multiple communication and sensing sectors. The directional sensing and communication models are presented below.

### 3.1. Directional Sensing Model

**Figure 1.**
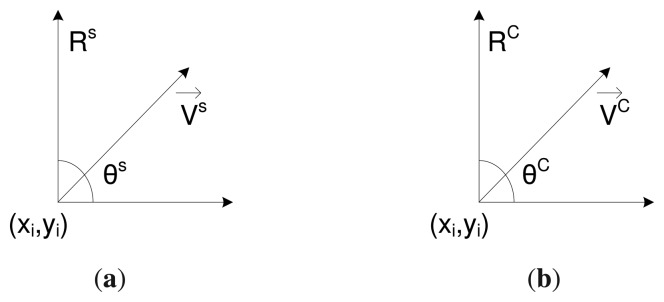
The directional sensing and communication models. (**a**) Sensing model; (**b**) communication model.

We assume a directional sensing model in which the sensing area of a sensor is a sector, denned by three-tuple < *R^S^*,*V→^S^*,*θ^S^* >, where, *R^s^* is the sensing radius, *V→^s^* is the directional vector that represents the center line of the sensing sector and *θ^s^* is the angle of the field of view, as shown in Figure 1a.

We also assume that each node's sensing region is divided into several sectors, ranging from 2 to 6, and the sensing range of each sector is identical. The velocity of a moving target is less than the sensor's sensing frequency. The assumption is relevant, because the minimum sensing interval for an ultrasonic sensor is usually around 10^−2^ to 10^−3^ s and that of an infrared sensor is usually above 10^−4^ s, which is much higher than the target's possible velocity [34,35]. We also assume that the CH can wake up any of its member nodes on-demand and the time required (The wake up time for a typical sensor mote is 6 *μs* [36]. Thus, sum of the communication, computation and wake up time does not cross 1 ms.) is much less than the time in which a high speed target can travel one meter.

### 3.2. Directional Communication Model

The communication model of each sensor is also defined by three-tuples < *R^c^*,*V→^c^*, *θ^c^* >, where, *R^c^* is the communication radius, *R^c^* ≥ 2 × *R^s^, V→^c^* is the directional vector represents the center line of the communication sector and *θ^c^* is the angle of the field of view (FOV), as shown in Figure 1b. Like in the sensing model, a node can communicate in multiple sectors, ranging from 2 to 6, and the communication range of each sector is identical. We also assume that, at a certain time, the sensing and communication sectors of a node may be the same or different, determined by the cluster formation and sensing coverage algorithms. The notations used throughout this paper are enlisted in Table 1.

## 4. MTDC Architecture

The proposed moving target tracking through distributed clustering (MTDC) mechanism has the following design components: cluster formation algorithm, gateway node selection mechanism, determination of active sensing nodes and their sensing directions and the cluster head-based target tracking algorithm. In what follows, we describe in detail the operations of the aforementioned components.

### 4.1. Cluster Formation

The philosophy of our proposed MTDC cluster formation algorithm is as follows. It must achieve the following three goals: to ensure balanced energy consumption among the network nodes so that the network lifetime is maximized, to increase the number of members in each cluster so that the total number of clusters formed in the network is reduced and to reduce the number of hops required to deliver data packets to the sink from the cluster heads. Therefore, we develop an integrated metric through a linear combination of three sub-metrics: residual energy, number of neighbors and distance to the sink.

The cluster formation starts just after deployment of the network nodes. As described in Section 3, each node knows the ID, residual energy and (*x*,*y*) location of its neighbor nodes through the neighborhood discovery algorithm. Thus, each node can calculate the number of neighbors |*n_i,s_*| it has, in each sector *s* ∈ ψ*_c_* and its distance from the sink, *d*(*i*, *sink*). At first, each node *i* ∈ 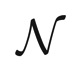 calculates the cluster formation weight for itself in sector *s* ∈ ψ*_c_*, (*W_i,s_*) and all of its neighbor nodes, as follows,

(1) $$\begin{array}{cc} W_{i,s}=w_{1}\times \frac{E_{\text{res}}^{i}}{E_{\text{init}}^{i}}+w_{2}\times \frac{\left|n_{i,s}\right|}{n_{\text{max}}^{i}}+w_{3}\times \left\{1-\frac{d\left(i,\text{sink}\right)}{d_{\text{max}}}\right\}, & \forall s\in \psi_{c} \end{array}$$

where *w*_1_, *w*_2_**, and *w*_3_ are the weight factors, *w*_1_ > *w*_2_ > *w*_3_ and *w*_1_ + *w*_2_ + *w*_3_ = 1; the $n_{\text{max}}^{i}$ is the maximum number of neighbor nodes any sensor *i* has, and it is determined as follows,

(2) $$n_{\text{max}}^{i}=\underset{\forall s\in \psi_{c}}{\max}\left\{\underset{\forall j\in n_{i,s}}{\max}\left\{\underset{s\in \psi_{c}}{\max}\left|n_{j,s}\right|\right\},\left|n_{i,s}\right|\right\}$$

Then, each node *i* ∈ 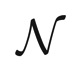 checks the following condition,

(3) $$\underset{\forall s\in \psi_{c}}{\max}\left(W_{i,s}\right)\geq \underset{\forall s\in \psi_{c}}{\max}\left\{\underset{\forall j\in n_{i,s}}{\max}\left\{\underset{s\in \psi_{c}}{\max}\left(W_{j,s}\right)\right\}\right\}$$

If Equation (3) returns as true, for any node *i* ∈ 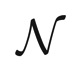, it declares itself as the cluster head (CH).

Therefore, Equation (1) ensures that, at each neighborhood environment, the node that has the highest *W* value is chosen as the CH. Note also that the CH selection metric is calculated as the weighted linear combination of three sub-metrics: residual energy, number of neighbor nodes and distance from the sink with weight factors *w*_1_, *w*_2_ and *w*_3_, respectively. The residual energy is given the highest weight (*w*_1_), while the distance factor is given the lowest (*w*_3_)*.* The first term helps to ensure the balanced energy consumption among the network nodes, while the second term reduces the number of clusters formed in the network. Finally, the third term reduces the number of hops required to deliver the sensed data packets to the sink. Thus, a node having a higher amount of residual energy, a higher number of neighbor nodes and reduced or less distance from the sink will get higher priority to be selected as the CH. More explicitly, among two or more nodes having a similar distance from the sink and the same number of neighbor nodes, the one having higher residual energy will be selected as the CH. In summary, Equation (1) trades-off among the three sub-metrics for achieving the goals of efficient moving target tracking in DSNs.

The CH calculates the working communication sector of the CH using the direction towards the sink, which is chosen as the working communication sector. Each cluster head sends a cluster member request CH_REQUEST message to all of its neighbors, which contains: (1) the CH ID; (2) the working communication sector ID; and (3) the set of neighbors in the working communication sector.

If a neighbor node *j* ∈ *n_CH,s_* receives the CH_REQUEST message from a CH (*i.e.*, the node is in the working communication sector of the CH), then the node *j* sets its communication direction facing towards the CH. Then, it replies to CH with the cluster member confirm CH_CONFIRM message that consists of: (1) the node ID; and (2) the CH ID. After that, it updates *W* for itself and its neighbors in all sectors using Equation (1). If a node receives more than one cluster member CH_REQUEST message from different cluster heads, then it joins the cluster that is the closest.

**Figure 2.**
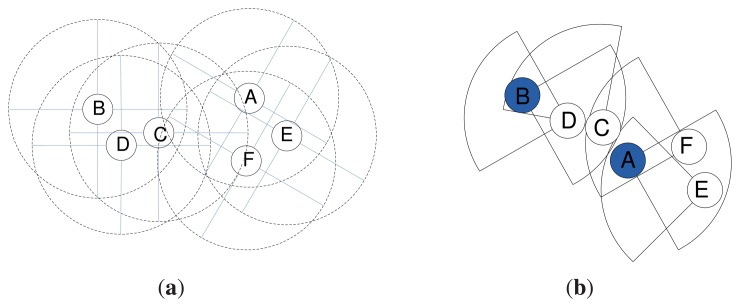
Cluster formation. (**a**) Before cluster formation; (**b**) after cluster formation.

The cluster formation procedure is presented in Algorithm 1. An example of cluster formation is depicted in Figure 2, where sensor Bhas the maximum W and is elected as the cluster head that forms a cluster with members *C* and *D*. Similarly, node Acreates a cluster with member nodes *E* and *F*.



**Algorithm 1** Cluster formation algorithm, at each sensor node *i* ∈ 

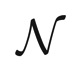
.
1.*n_i,s_* ← set of neighbor nodes of node *i* in sector *s*2.**while** TRUE **do**3. **if**
Eq 3 returns TRUE **then**4.  *CH* ← *i*5.  *o* ← orientation of node *i* that has the highest *W*6.  Node *i* sets *o* as its working communication sector7.  CH sends CH_REQUEST to all *j* ∈ *n_i,s_*, ∀ *_s_* ∈ ψ*_c_*8. **else if** Node *i* receives CH_REQUEST from any CH **then**9.  *p* ← Working communication sector of CH10.  **if** Node *i* is NOT a member of any other CHs **then**11.   *q* ← orientation of *i* facing towards CH12.   Node *i* sets *q* as its working communication sector13.   Node *i* sends CH_CONFIRM message to the CH14.  **end if**15. **end if**16. Update the *n_i,s_* and *W_i,s_*17.**end while**


### 4.2. Gateway Selection

After the cluster formation has been completed, gateway nodes are selected for data communication among the clusters. The gateway (GW) nodes help to develop a network backbone for data communication. Gateway nodes are selected by the cluster head to communicate with other clusters. A sensor *i* can be considered as a candidate gateway node of the CH if it can directly communicate with the nearby cluster head or through another member of a nearby cluster. We assume that the reception antenna is omnidirectional. A CH computes the gateway selection weight (G) for all candidate gateway nodes as follows,

(4) $$\begin{array}{cc} G_{i,s}=w_{1}\times \frac{E_{\text{res}}^{i}}{E_{\text{init}}^{i}}+w_{2}\times \frac{\left|n_{i,s}^{o}\right|}{n_{\text{max}}^{i}}+w_{3}\times \left\{1-\frac{d\left(i,\text{sink}\right)}{d_{\text{max}}}\right\}, & \forall s\in \psi_{c} \end{array}$$

where *w*_1_, *w*_2_, and *w*_3_ are the weight factors, *w*_1_ > *w*_2_ > *w*_3_ and *w*_1_ + *w*_2_ + *w*_3_ = 1. Now, a CH selects a sensor node *i* as a gateway node if and only if the node satisfies the following condition,

(5) $$\underset{\forall s\in \psi_{c}}{\max}\left(G_{i,s}\right)\geq \underset{\forall j,S_{j}\in n_{k,wcs,}j\neq i}{\max}\left\{\underset{s\in \psi_{c}}{\max}\left(G_{j,s}\right)\right\}$$

Therefore, the CH selects a node *i* as the gateway that has the highest *G* value using one-hop neighborhood information, and it requires light weight computations. Then, the CH *k* broadcasts a gateway request GW_REQUEST message consisting of the: (1) gateway node ID; and (2) gateway working communication sector ID; thus, the gateway and all other cluster member nodes come to know this selection. The selected gateway sends a gateway confirmation GW_CONFIRM message to the other cluster head.

**Figure 3.**
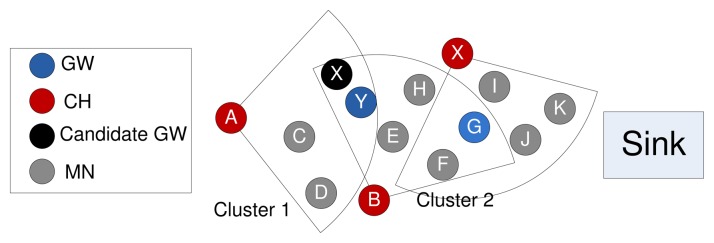
Gateway node selection.

An example gateway selection procedure is presented in Figure 3. Here, *A* and *B* are the cluster heads of Clusters 1 and 2, accordingly. The member nodes *X* and *Y* are common in both of the clusters, so these two nodes are considered as the candidate gateway nodes. Node *Y* has the highest G compared to all of the candidate gateway nodes. Therefore, *Y* is selected as the gateway node to communicate with the next-hop cluster.

### 4.3. Cluster Area Coverage

Once the communication backbone is constructed, each cluster head selects some active nodes, which will remain awake initially. The other nodes of the cluster will be in sleep mode to conserve energy. A greedy approach is used in the selection of the active nodes to cover the border area of a cluster, as well as the middle area.

#### 4.3.1. Border Area Coverage

Since there is a high probability of having overlapped regions covered by directional sensor nodes, our mechanism targets minimizing the number of active sensor nodes by reducing duplicate coverage and covering the cluster border area only. Since the CH is aware of the locations of its member nodes, it can determine the set of sensor nodes *B_CH_* that are candidate nodes for border area coverage. Note that each node *i* ∈ *B_CH_* must satisfy the following condition,

(6) $$\min\left\{d\left(i,x\right)\right\}<R_{s}$$

where *x* is any point on the border arc or straight line. The condition 6 implies that at least one sector *s* ∈ ψ*_s_* of node *i* ∈*B_CH_* can cover some area of the cluster border. Therefore, our problem now boils down to the selection of nodes from *B_CH_* in such a way that the cluster border area can be covered with the minimum number of nodes, which is an NP-complete problem [22]. Thus, we develop a greedy solution for the problem.

**Figure 4.**
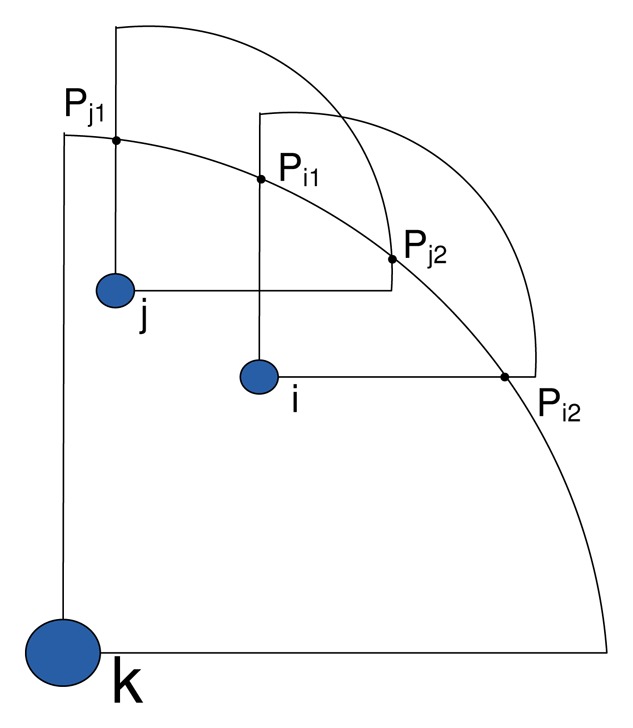
Border area coverage overlapping.

At the first step, the CH finds all *i* ∈ *B_CH_* nodes whose coverage area has no overlapping region with any other neighboring nodes, denoted by *ζ_B_*, using Equation (7), as follows,

(7) $$\zeta_{B}=\left\{\begin{array}{cc} i|\text{overlap}\left(i,j\right)=0, & \forall j\in B_{CH},\;j\neq i,\forall s\in \psi_{s} \end{array}\right\}$$

where the function *overlap*(*i*,*j*) returns zero if node *i* has no overlap with any of its neighboring nodes *j* G *B_CH_*, and one otherwise. As shown in Figure 4, node *k* is the CH, node *j* intersects the cluster border on points (*x*_1_*_j_*,*y*_1_*_j_*) and (*x*_2_*_j_*,*y*_2_*_j_*) and node *i* intersects the cluster border on points (*x*_1_*_i_*,*y*_1_*_i_*) and (*x*_2_*_i_*,*y*_2_*_i_*). Node *i* has overlapping coverage with node *j.* Therefore, the *overlap*(*i*,*j*) function can perform the test using condition 8 and returns the result accordingly.

(8) $$\left(x_{1j}<x_{1i}∥x_{2i}<x_{2j}\right)\&\&\left(y_{2j}<y_{1i}∥y_{2i}<y_{2j}\right)$$

After getting the result, the CH activates all of the nodes having no overlap with any other nodes and puts them into a list *ζ_B_*, and these nodes are activated.

At the second step, the MTDC border area coverage mechanism uses a greedy approach to activate the nodes based on their area coverage. The CH sorts the remaining *B_cov_* = {*B_CH_* \ *ζ_B_*}border area sensors in descending order of the maximum border lengths that they cover, which is computed using Equation (9),

(9) $$\begin{array}{cc} \underset{\forall s\in \psi_{s}}{\max}\left\{\text{len}\left(i,s\right)\right\}, & i\in B_{\text{cov}} \end{array}$$

where function *len*(*i*, *s*) returns the length of the border covered by any node *i* ∈ *B_cov_* in sector *s* ∈ ψ*_s_*. The CH then activates the first node *i* ∈ *B_cov_* and migrates it to a separate list of active nodes, *B_active_*. Before activating the second node *j* ∈ *B_cov_*, the CH compares if the node *j* has less than *a*% overlap with *i* and migrates it to *B_active_*, if the test returns true; otherwise, node *j* is not activated. This process continues till the complete border of the CH is covered. However, if the complete border is not covered in the first round, the same procedure will be executed iteratively with the increased value of *α* (e.g., *α* = 2 × *α*) in each iteration. The cluster border area coverage procedure is summarized in Algorithm 2.

**Algorithm 2** Border area coverage algorithm, at each cluster head (CH).
**INPUT:**
*B_CH_***OUTPUT:**
*B_active_*1.**for all**
*i* ∈ *B_CH_* do2. Develop *ζ_B_*, the set of all nodes having no overlapping region with neighbors, using Equation (7)3.**end for**4.*B_cov_* ← {*B_CH_* \ *ζ_B_*}5.**sort**
*B_cov_* in descending order of covered border length6.*B_active_* ← first element in *B_cov_*7.**while** Complete border is not covered **do**8. **for all**
*k* ∈ *B_cov_*
**do**9.  **for all**
*i* ∈ *B_active_* && *i* ≠ *k*
**do**10.   **if**
*overlap*(*i*, *k*) < *α*
**then**11.    *B_active_* ← {*B_active_* ∪ *i*}12.    *B_cov_* ← {*B_cov_* \ *i*}13.   **end if**14.  **end for**15. **end for**16. *α* = *α* × 217.**end while**18.*B_active_* ← {*ζ_B_* ∪ *B_active_*}


#### 4.3.2. Middle Area Coverage

Note that the border area coverage algorithm activates the sensor nodes that can detect a target moving in or out of the border area of a CH. However, these sensors fail to track the path of a moving target inside the CH area. Therefore, we activate a minimum number of sensors in the middle area to increase the target tracking accuracy using a similar procedure stated in Algorithm 2.

First, we find a set of candidate sensor nodes for middle area coverage for a given cluster head, *M_CH_* = {*M_CH_*\ *B_active_*}, where *M_CH_* is the set of all members of the CH. Then, we find all *i* ∈ *M_CH_* nodes whose coverage area has no overlapping region with any other neighboring nodes, denoted by *ζ_M_*, using Equation (10), as follows,

(10) $$\zeta_{M}=\left\{\begin{array}{cc} i|\Lambda \left(i,j\right)=0, & \forall j\in M_{CH},\;j\neq i,\forall s\in \psi_{s} \end{array}\right\}$$

where Λ(*i*, *j*) is a function of calculating the amount of overlapped area between nodes *i* and *j*, which is elaborated in Section 4.3.4.

In the second step, the proposed MTDC middle area coverage algorithm finds the sorted list of *M_cov_* = {*M_CH_* \ *ζ_m_*} in ascending order of their amount of overlapped area coverage with the neighborhood nodes. The CH then activates the first node *i* ∈ *M_cov_* and migrates it to a separate list of active nodes, *M_active_*. Therefore, the same procedure is applied to activate the rest of the nodes from *M_cov_* as used in the border area coverage algorithm. The steps of activating the sensor nodes for middle area coverage are summarized in Algorithm 3.

**Algorithm 3** Middle area coverage algorithm, at each CH.
**INPUT:**
*M_CH_*,*B_active_***OUTPUT:**
*M_active_*1.**for all**
*i* ∈ *M_CH_*
**do**2. Develop *ζ_M_*, the set of all nodes having no overlapping region with neighbors, using Equation (7)3.**end for**4.*M_cov_*←{*M_CH_*\*ζ_M_*}5.**sort**
*M_cov_* in ascending order of amount of overlapped area6.*M_active_* ← first element in *M_cov_*7.**while** Complete middle area is not covered **do**8. **for all**
*k* ∈ *M_cov_* do9.  **for all**
*i* ∈ (*M_active_* ∪ *B_active_*) && *i* ≠ *k*
**do**10.   **if** Λ(*i*,*k*) < *α* then11.    *M_active_* ← {*M_active_* ∪ *i*}12.    *M_cov_* ← {*M_cov_*\ *i*}13.   **end if**14.  **end for**15. **end for**16. *α* = *α* × 217.**end while**18.*M_active_* ← {ζ_*M*_ ∪ *M_active_*}


#### 4.3.3. On-Demand Node Activation

Note that the aforementioned border and middle area coverage algorithms guarantee that a moving target will be detected by at least one of the member sensing nodes of the visiting CH. In the literature, this is known as a *κ*-coverage solution, and in this case, *κ* = 1. However, our proposed MTDC allows CHs to activate more sensing nodes dynamically, when a target is detected, to increase the target tracking accuracy. The set of candidate sleeping nodes that can be activated for increasing the coverage is $M_{CH}^{\prime }=\left\{M_{CH}\backslash B_{\text{active}}\backslash M_{\text{active}}\right\}$. When a CH receives the target detection sensing information from any member node *i*, it first develops a set of sleeping nodes that can cover the active sensing sector of *i*, as follows,

(11) $$D_{\text{cov}}^{i}=\left\{\begin{array}{cc} j|\Lambda \left(i,j\right)>0, & \forall j\in M_{\text{CH}}^{\prime },\;j\neq i,\forall s\in \psi_{s} \end{array}\right\}$$

Now, the CH sorts the elements of *D_cov_* in descending order of the amount of coverage area overlapped with node *i.* Then, nodes from the set *D_cov_* are activated one after another, so that each point in the active sector of *i* is covered by at least *κ* sensors. The steps of the on-demand node activation mechanism have been summarized in Algorithm 4.

Similarly, when sensor node *i* notifies the CH that the target has gone out of its coverage area, the newly-activated nodes will be sent to sleeping mode again. Thus, our MTDC algorithm activates the sleeping nodes on-demand for a short period of time, so that the target tracking accuracy can be enhanced.

**Algorithm 4** On-demand node activation algorithm, at each CH.
**INPUT:**
*M_CH_*, *B_active_*, *M_acttve_*, *κ***OUTPUT:**
$D_{\text{active}}^{i}$1.
$M_{CH}^{\prime }\leftarrow \left\{M_{CH}\backslash B_{\text{active}}\backslash M_{\text{active}}\right\}$2.Find 
$D_{\text{cov}}^{i}$ using Equation (11)3.**sort**
$D_{\text{cov}}^{i}$ in descending order of amount of Λ(*i*, *j*)4.
$D_{\text{active}}^{i}\leftarrow \text{first element in}D_{\text{cov}}^{i}$5.**while** each point of *i*'s sector is not *κ*-covered **do**6. 
$m\leftarrow D_{\text{cov}}^{i}$7. 
$D_{\text{active}}^{i}\leftarrow \left\{D_{\text{active}}^{i}\cup m\right\}$8. 
$D_{\text{cov}}^{i}\leftarrow \left\{D_{\text{cov}}^{i}\backslash m\right\}$9.**end while**


#### 4.3.4. Overlapping Coverage Area Calculation

**Figure 5.**
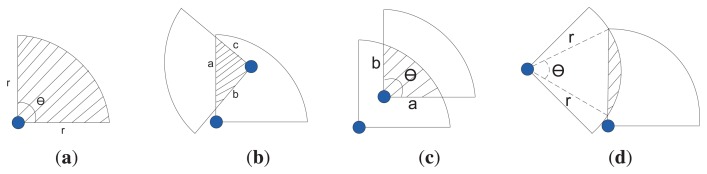
Different types of overlapping. (**a**) Candidate sector; (**b**) Case 1; (**c**) Case 2; (**d**) Case 3.

As discussed before, when a CH attempts to activate a sensor node, it needs to calculate the amount of area covered by multiple active sensor nodes, *i.e.*, the amount of overlapped area, denoted by Λ. Two sensing nodes may overlap each other in many different ways, and they can be broadly categorized into three different cases, as shown in Figure 5. In Case 1, the overlapping area is covered by three straight lines; in Case 2, the area is covered by two straight lines and one arc; and in Case 3, the overlapping region is covered by one straight line and one arc.

Now, using simple geometry [37], we can calculate the area of a candidate sector that has no overlapping area (Figure 5a) bounded by Case 1, Case 2 and Case 3 using Equations (12) and (14)—(16), respectively.

(12) $$\Lambda =\frac{r^{2}\theta }{2}$$

(13) $$s=\frac{a+b+c}{2}$$

(14) $$\Lambda =\sqrt{s\left(s-a\right)\left(s-b\right)\left(s-c\right)}$$

(15) $$\Lambda =\frac{ab}{2}\left[\theta -\tan^{-1}\left(\frac{\left(b-a\right)\sin\left(2\theta \right)}{\left(b+a\right)+\left(b-a\right)\cos\left(2\theta \right)}\right)\right]$$

(16) $$\Lambda =\frac{r^{2}}{2}\left(\theta -\sin\theta \right)$$

In addition to the above cases, we may encounter situations where the overlapping area has two arcs and one straight line, or two arcs and two straight lines, or two arcs only, or four straight lines, *etc.* In these cases, we can divide the area into two or more separate parts, where each part falls into one of the aforementioned three cases. Thus, we can calculate the area of overlapping regions of any shape.

A node *i* can calculate the percentage of overlapped area coverage with any of its neighbor node *j* for any of its candidate sectors *s* as follows,

(17) $$\begin{array}{cc} \eta \left(i,j,s\right)=\frac{\Lambda \left(i,j\right)}{\Lambda_{i}}\times 100\%, & \forall j\in n_{i,s},\forall s\in \psi_{c} \end{array}$$

**Figure 6.**
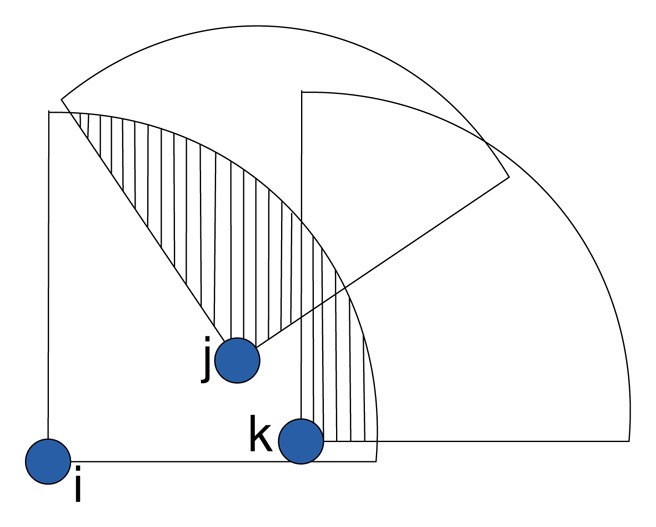
Coverage area overlapped by multiple nodes.

In the case, node *i* has overlapping coverage with more than one neighbor nodes, as shown in Figure 6, it can calculate the total amount of overlapping as follows,

(18) $$\eta \left(i,s\right)=\overset{\left|n_{i,s}\right|}{\underset{j=1}{\text{∑}}}\eta_{i,j}^{s}$$

Thus, we can calculate the area of the overlapping region and the percentage of overlap for any sensor member node of a CH.

### 4.4. Target Tracking

In this section, we describe how a CH determines the location of a target by exploiting sensed data from its member nodes. When a target is detected by a sensor node, it sends the sensing data, < NodeID, sector, DateTime>, to the CH. For a moving target, the CH will receive such sensing information from many of its member nodes. Then, the CH combines all of the sensing information to determine the arc in which the target is crossing, described as follows.

The CH takes the initial angle λ as the full sector of the first member node that senses the target. Then, it reduces the angle size by taking INTERSECTION of coverage of all other nodes that sense the target using Equation (19). If a sensing node has an overlapping coverage area with the first node, but the former does not report any sensing information, the CH combines the corresponding central angles by the MINUS operation using Equation (20). Therefore, the arc corresponding to the resultant angle λ′ is denoted as the location in which the target passed. Therefore, the target tracking accuracy increases with the number of sensing nodes that report sensing information to the CH.

(19) $$\lambda^{\prime }=\lambda \underset{m\in \text{IN}}{\cap }{\text{Angle}}_{m}$$

(20) $$\lambda^{\prime }=\lambda \backslash_{m\in \text{OUT}}{\text{Angle}}_{m}$$

here, *IN* is the set of member nodes indicating the target's presence and *OUT* is the set of members indicating the target's absence.

**Figure 7.**
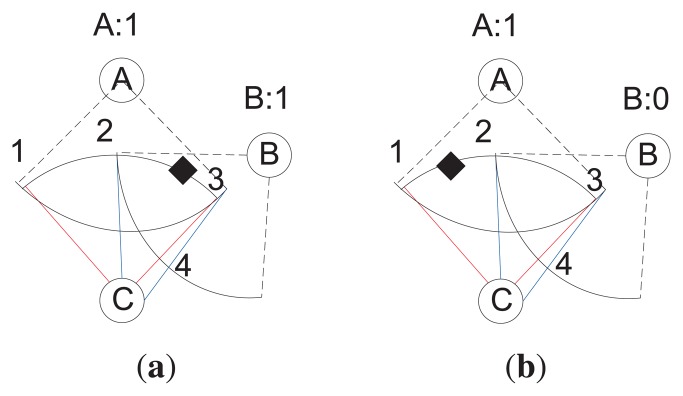
Determination of a target's location.

Figure 7 illustrates the target tracking example. In Figure 7a, node *C* senses the targets presence first, and *B* and *A* also sense the presence of the target and send the information to the CH. The CH calculates the angle ∠ 1*C*3 using intersection Points 1 and 3 of node *A* and *C* and ∠2*C*4 using intersection Points 2 and 4 of node *B*. Then, it performs the INTERSECTION operation on the angles and gets the resultant angle (λ′) ∠2*C*3. The corresponding arc of ∠2*C*3 is “23”. Therefore, the arc “23” is estimated as the location of the target. Thus, if more sensors detect the target, the arc length can further be reduced.

In Figure 7b, node *C* and *A* sense the target's presence and *B* cannot sense the presence of the target. The CH calculates the angle ∠1*C*3 using intersection Points 1 and 3 of node *A* and *C* and ∠2*C*4 using intersection Points 2 and 4 of node *B.* Then, it performs the MINUS operation on the angles and gets the resultant angle ∠1*C*2. The corresponding arc of ∠1*C*2 is “12”, and it is the estimated location of the target.

### 4.5. Data Reporting

Data packets are sent to the sink using inter-cluster communication through the gateway nodes. Data transmission from the CHs to the sink is event-triggered in our MTDC system, *i.e.*, after receiving sensed data packets that contain the sensor ID and timestamp of the event, a CH forwards them toward the sink. The target's movement from one location to another causes new sensor nodes to wake up, and thus, many sensed data packets are received by the corresponding CHs. Thus, the data communication is triggered by the events occurring in the terrain.

In a sensor network, a significant amount of energy is spent due to the transmission and reception of data packets. Therefore, data processing and aggregation at the CHs before forwarding it to the sink has proven to provide better performance [38,39]. The cluster head-based solution of MTDC gives it the opportunity to diminish redundant data reporting to the sink through data integration, decreasing the energy consumption of battery-powered nodes.

### 4.6. Cluster Management

Cluster management is required to reorganize the cluster head nodes periodically, so that one node does not run out of energy, reducing the network lifetime. The cluster management procedure consists of three phases: cluster head re-election, member node re-selection and gateway node renewing.

When the residual energy of the CH becomes less than a predefined threshold *γ^th^*, then the CH collects information from its neighbors to elect a new cluster head using the same procedure stated in Section 4.1. Then, the old CH becomes an ordinary node, and it doubles the energy threshold value so that the possibility of selecting the same node as the cluster head is decreased, and the energy load distribution becomes more balanced across the network.

Similarly, when the residual energy of a member node or a gateway node becomes less than the predefined threshold *γ^th^*, then it notifies the CH, and a new member or a new gateway is selected by the CH using the same procedure stated before.

### 4.7. Discussion

Note that, in MTDC, the weighted linear combination of three sub-metrics (residual energy, number of neighbor nodes and distance from the sink of a node) produces an integrated metric, which has been used to select CHs and gateways in the network. The selection of CHs exploits single-hop neighborhood information only. The computation and communication overheads of moving target tracking are placed on CHs. Therefore, the MTDC is a distributed solution and expected to provide satisfactory performance for increasing network size.

However, the key limitation of this work is the lack of mathematical expressions for the appropriate values of weight factors *w*_1_, *w*_2_ and *w*_3_ used in Equations (1) and (4). The optimal values of them are impacted by the network node density, initial node energy, network size and shape. The choice of sub-optimal values of the weight factors might reduce the performance of the proposed MTDC system. The simulation experiment-based determination of their values in our current work is the first research step, and it does not guarantee the optimal choice. A dynamic selection technique might further improve the MTDC performance. The analytical modeling to dynamically select the optimal values of the weight factors has been left for future work.

## 5. Performance Evaluation

In this section, we study the comparative performances of the real-time distributed target tracking (RDTT) [8], adaptive basis algorithm (ABA) [19] and the proposed moving target tracking through distributed clustering (MTDC) mechanism in terms of target tracking accuracy, network lifetime, the standard deviation of residual energy and protocol operation overhead.

### 5.1. Simulation Environment

We evaluate the performances of the studied target tracking mechanisms in ns-3, a discrete-event network simulator [21]. As sensor nodes, we use the ns-3 StaWifiMac model, and the nodes use the ConstantPositionMobility model and the target use RandomDirection2dMobility model. The RandomDirection2dMobility model use defined values for speed and random values for pause time, direction and acceleration of the moving target, which corresponds to the real-life scenario. For setting the channel properties, such as the delay loss model, propagation delay model, data rate and channel characteristics are defined using the YansWifiPhy channel model. We also use a station manager model for packet management that enables fragmentation for very large packets. We employ RCRT [40] as a transport protocol that ensures end-to-end reliable and energy-efficient data transfer.

We deploy the sensors and the moving targets uniformly in a region of 1000 × 1000 *m*^2^. We run simulation for 1000 s. 600 stationary sensor nodes and a few moving targets are considered. We have used the following values for the weight factors, *w*_1_ = 0.45, *w*_2_ = 0.35, and *w*_3_ = 0.20, determined through numerous simulation runs for different network size, node density, and initial node energy values as in [41,42]. The network configuration parameters are shown in Table 2. For each graph points, we run 10 simulation runs and take the average of the results.

### 5.2. Performance Metrics

The comparative performances of our proposed MTDC algorithm with those of RDTT [8] and ABA [19] have been carried out for varying number of sensing and communication sectors, number of sensor nodes deployed in the terrain, moving velocity of targets, number of targets moving in the terrain, etc. The following performance matrices are evaluated for comparison.

- Target tracking accuracy: The target tracking accuracy percentage is measured as the deviation percentage of the detected path from the actual path, the ratio of which is the actual path to the detected path of the target. A lesser value means that the detected path is less diverted from the actual path. Therefore, the lesser the value is, the more accurate is the tracking.
- Number of active sensing nodes: The active sensing nodes that are required to cover all of the targets entering and exiting in the terrain are measured. The lesser the value is, the more is the number of sensor nodes that go into sleep mode and conserve energy.
- Standard deviation of residual energy: The standard deviation of energy defines the average variance between the residual energy levels for all nodes and is measured by Equation (21),
  (21) $$\sigma =\sqrt{\frac{1}{\left|\text{N}\right|}\overset{\left|\text{N}\right|}{\underset{i=1}{\text{∑}}}{\left(E_{i}-\mu \right)}^{2}}$$
  where *E_i_* and *μ*, are, respectively, the residual energy of node *S_i_* and the mean residual energy for all nodes. Therefore, the value of *σ* indicates how well the energy consumption is distributed among the sensor nodes. The smaller the value, the better is the capability of the MTDC system to balance the energy consumption.
- Network lifetime: We measure the network lifetime during the entire process. A greater amount of time corresponds to better performance.
- Tracking operation overhead: The tracking operation overhead is denned as the ratio of the total number of control bytes (due to REQUEST, CONFIRM, RTS, CTS, ACK, *etc.* messages) transferred during the simulation period to the total number of data bytes received by the sink. A smaller percentage of overhead describes better performance.

### 5.3. Simulation Results

To evaluate the robustness of our proposed MTDC mechanism in different environments, we study the performances for varying numbers of sensor nodes deployed in the network and the number of sensing and communication sectors.

#### 5.3.1. Impacts of the Number of Sensor Nodes

The performance metrics discussed before are measured for varying numbers of directional sensor nodes ranging from 200 to 1000, and the number of sectors is kept at four.

**Figure 8.**
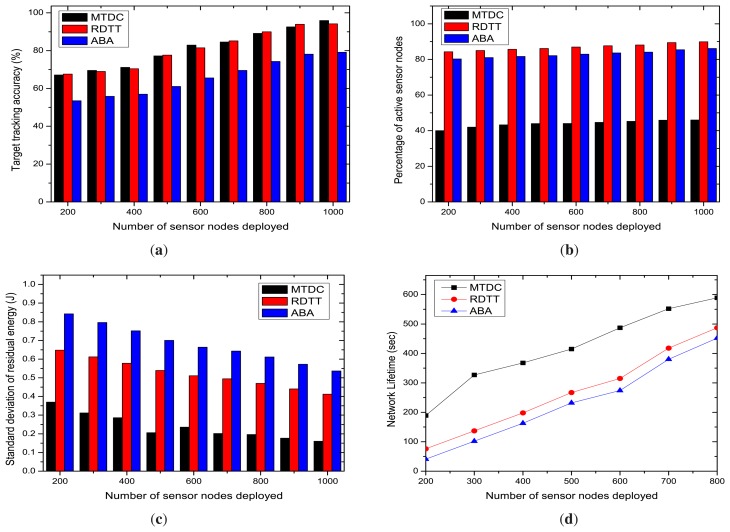
Impacts of the number of sensor nodes. (**a**) Target tracking accuracy; (**b**) percentage of active sensing nodes; (**c**) standard deviation of residual energy; (**d**) network Lifetime.

Figure 8a reveals a substantial improvement in terms of target tracking accuracy, which is measured from the deviation of the detected path of a target from the actual path. The accuracy percentage has been achieved by our MTDC algorithm, and compared to RDTT and ABA, it is relatively higher. Our algorithm shows better performance, because the CH takes the responsibility of being a coordinator, aggregates the target tracking information from the member sensor nodes and detects the best possible path without any loss of information. The graphs in Figure 8a show that for both algorithms, the target tracking accuracy percentage increases as the number of sensor nodes deployed increases. This is because, when there are many nodes, the target will be tracked by a greater number of nodes and accuracy percentage will go higher.

The graphs in Figure 8b show that, initially, the number of active sensor nodes is almost the same with the varying number of deployed sensor nodes in MTDC, ABA and RDTT. In RDTT and ABA, the number of active nodes is much higher compared to our MTDC algorithm. This is the most important achievement of our MTDC algorithm, and it is due to the execution of our proposed algorithm at the CHs, not at individual sensor nodes. The CHs are performing as controllers for determining active sensors and their sensing directions; thus, more optimal decisions are made to send many overlapping sensors into sleep mode. On the contrary, in the RDTT algorithm, individual sensor nodes run the target tracking algorithm, so it has poor coordination among the nodes and is unable to implement an optimal area coverage. In ABA, the number of active sensor nodes are lower than RDTT; this is because a few nodes are used for tracking objects only when the object crosses its line of sight. Thus, it does not cover the whole area. Therefore, a substantial performance improvement by our MTDC system has been achieved.

Figure 8c shows the standard deviation of residual energy levels for increasing the number of sensor nodes deployed in the network. The graphs depict that the standard deviation of the residual energy of nodes decreases with the increasing number of nodes deployed in the network. Our proposed MTDC algorithm gives better performance than RDTT and ABA. This happens because RDTT and ABA do not consider the residual energy level of nodes when selecting CHs, gateways and active sensing nodes, and thus, this increases unbalanced energy consumption. Furthermore, updating the residual energy thresholds for selecting/renewing CHs in our MTDC system ensures balanced energy consumption.

The comparison of network lifetime offered by the MTDC, RDTT and ABA algorithms is shown in Figure 8d. As expected theoretically, the network lifetime linearly increases with the number of additional sensors deployed in the network for all of the studied protocols. Our MTDC system achieves better lifetime compared to the RDTT and ABA algorithms, because MTDC uses a clustering approach to reduce the network overhead and area coverage algorithms for activating a few nodes initially, which enhance the network lifetime.

#### 5.3.2. Impacts of the Number of Sectors

In this section, we evaluate the impacts of the number of communication and sensing sectors, ranging from 2 to 6, on the performances of the studied algorithms. In this experiment, we have fixed the number of sensor nodes deployed in the area at 600.

**Figure 9.**
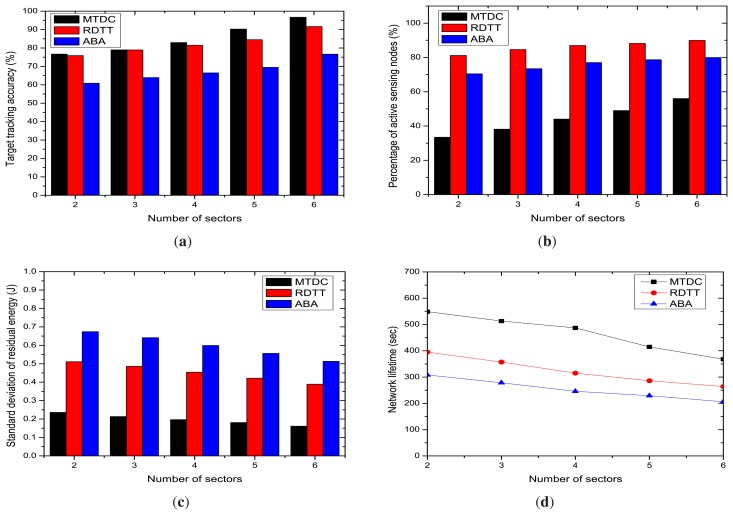
Impacts of the number of sectors. (**a**) Target tracking accuracy; (**b**) percentage of active sensing nodes; (**c**) standard deviation of the residual energy; (**d**) network Lifetime.

Figure 9a states that the target tracking accuracy percentage in MTDC, RDTT and ABA increases with the number of sensing sectors. The high number of sectors means a shorter arc length. Therefore, the target's path can be detected more accurately with a lower deviation from the actual path. The graphs also state that our MTDC algorithm performs better than the RDTT and ABA algorithms despite the increasing number of sectors. In our MTDC system, the CHs run the target coverage algorithm that determines the active sensor nodes and their sensing directions, so that the accuracy percentage is higher.

The number of active sensor nodes is increased with the number of sectors, as depicted in Figure 9b. However, in our MTDC algorithm, fewer sensing nodes remain active compared to RDTT and ABA. This happens for the following reasons: cluster formation helps the sensor nodes be coordinated by the CHs, and CHs select some active sensor nodes, which can track the target's path initially. Besides, the on-demand node activation procedure helps to send a good number of sensor nodes into the sleep state, reducing the active sensing nodes.

The graphs in Figure 9c state that the standard deviation of the residual energy level decreases slowly with the increasing number of sectors for MTDC, RDTT and ABA. Our proposed MTDC system gives much better performance than the RDTT and ABA. As stated in Section 5.3.1 for the number of sensors, the larger number of sectors also increases the number of options from which the CH can choose regarding the nodes to activate and which to keep in sleep mode. Therefore, the energy will be conserved.

The comparison of the network lifetime offered by the MTDC, RDTT and ABA algorithms is shown in Figure 9d for an increasing number of sectors. The network lifetime linearly decreases with the number of sectors for all of the studied protocols, since it increases the probability of activating a large number of nodes initially.

#### 5.3.3. Impacts of Target Moving Velocity

**Figure 10.**
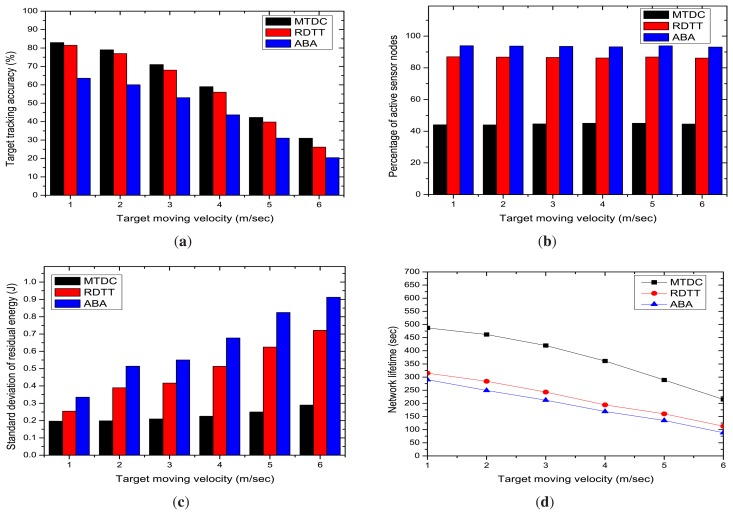
Impacts of target tracking velocity. (**a**) Target tracking accuracy; (**b**) percentage of active sensing nodes; (**c**) standard deviation of the residual energy; (**d**) network Lifetime.

In this section, we study the impacts of target moving velocity on the performances of the target tracking algorithms. Figure 10 states that the target tracking accuracy percentage in MTDC, RDTT and ABA decreases with the increasing target tracking velocity. The higher velocity of the target causes the sensor devices to miss data reporting, and thus, the accuracy decreases. The graphs also state that our MTDC algorithm performs slightly better than the RDTT and ABA algorithms irrespective of velocity levels. In our MTDC system, the CHs run the target coverage algorithm that determines the active sensor nodes and their sensing directions, so that the accuracy percentage is higher.

We also observe in Figure 10b that the target moving velocity has no impact on the percentage of active sensing nodes in the network, which is also expected theoretically. However, in our MTDC algorithm, fewer sensing nodes remains active compared to the RDTT and ABA algorithms, as stated in Section 5.3.2.

The standard deviation of residual energy linearly increases and the network lifetime decreases with the target moving velocity, as shown in Figure 10c,d, respectively. This is because when the target moving speed is high, node activation and deactivation happen more frequently. This causes more energy consumption and the rapid degradation of the network lifetime.

#### 5.3.4. Impacts of the Number of Moving Targets

In this section, we evaluate the impacts of the number of moving targets, ranging from 1 to 5, on the performances of the studied target tracking systems. In this experiment, we have fixed the number of sensor nodes deployed in the area at 600 and the number of sectors at four.

**Figure 11.**
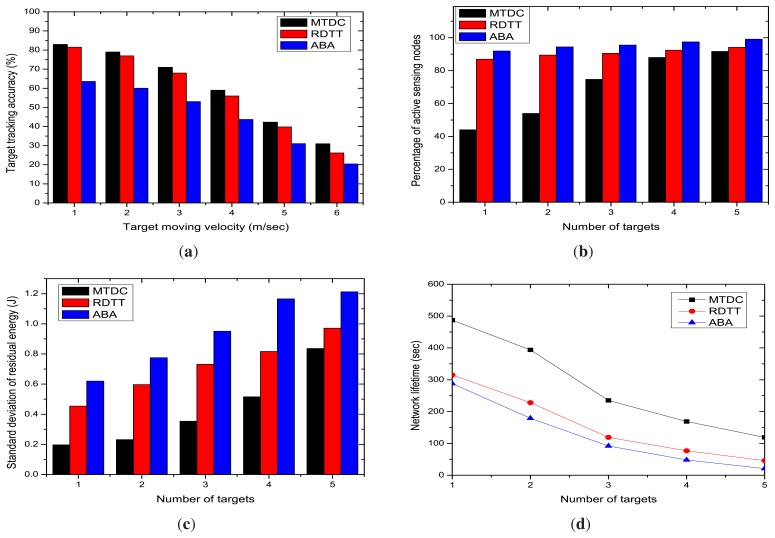
Impacts of the number of targets. (**a**) Target tracking accuracy; (**b**) percentage of active sensing nodes; (**c**) standard deviation of the residual energy; (**d**) network Lifetime.

Figure 11a depicts that the percentage of target tracking accuracy in MTDC, RDTT and ABA decreases with the increasing number of targets. When the number of targets in the vicinity increases, most of the sensor nodes become active and track them, and the communication overhead increases, which, in turn, decreases the accuracy. The graphs also state that our MTDC algorithm performs better than the RDTT and ABA algorithms. In our MTDC system, the CHs run the target coverage algorithm that determines the active sensor nodes and their sensing directions, so that the accuracy percentage is higher.

The number of active sensor nodes is increased with the number of moving targets, as depicted in Figure 11b. In our MTDC algorithm, fewer sensing nodes remain active compared to RDTT and ABA. Because the MTDC cluster formation mechanism selects a CH to coordinate the other nodes of the cluster, the on-demand node activation procedure can activate as few nodes as possible.

The standard deviation of residual energy linearly increases and the network lifetime decreases with varying the number of targets, as shown in Figure 11c,d, respectively. This is because, when the number of moving targets is higher, node activation and deactivation happen more frequently. This causes more energy consumption and the rapid degradation of the network lifetime.

#### 5.3.5. Tracking Operation Overhead

**Figure 12.**
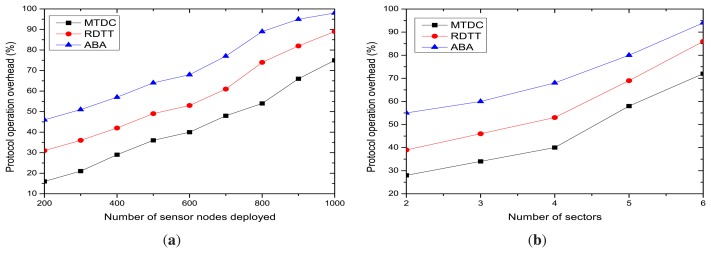
Tracking operation overhead. (**a**) Overhead *vs.* the number of nodes; (**b**) overhead *vs.* the number of sectors.

Finally, we measured the target tracking operation overhead for varying numbers of sensor nodes and sensing and communication sectors. Figure 12a,b, respectively, depicts that the tracking operation overhead linearly increases with both the number of sensor nodes and the number of sectors. This is because when the number of sensors and the number of sectors are increased, the number of active nodes is also increased, which, in turn, excels the communication overhead due to control packet transfer.

## 6. Conclusions

In this paper, we presented a distributed cluster-based moving target tracking system in a DSN. The cluster heads formed in the proposed MTDC system increase the target tracking accuracy through efficient aggregation of sensing data from member nodes. The CHs also reduce the amount of data packets transmitted toward the sink node; thus, they reduce the network bandwidth wastage, as well as increase the network lifetime. In MTDC, the CHs and the gateways are determined first; then, each CH addresses the area coverage problem by activating some border and middle area sensing nodes to conserve energy. This work has also increased the moving target tracking accuracy by on-demand activation of nodes. Our energy-efficient solution to the updating procedure of CHs, GWs and active sensor nodes is carried out by the cluster heads.

Although the proposed mechanism achieves better performance, further experimental and theoretical extensions are possible. As mentioned in Section 4.7, the weight factors in our system need a better mathematical analysis for dynamically selecting their values, and we have left this as future work.

## Figures and Tables

**Table 1.** List of notations.

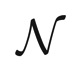	Set of all sensor nodes
*M_CH_*	Cluster member node
*d*(*i*, *j*)	Cartesian distance between nodes *i* and *j*
$n_{\text{max}}^{i}$	The max number of neighbors of any node *i* ∈ 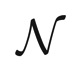 has
*d_max_*	The distance of the sink from the farthest node
Ψ*_c_*	The set of communication sectors of any node *i* ∈ 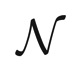
Ψ*_s_*	The set of sensing sectors of any node *i* ∈ 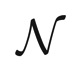
*n_i,s_*	The set of *i*'s neighbor nodes in sector *s* ∈ Ψ*_c_*
$E_{\text{init}}^{i}$	The initial energy of node *i*
$E_{\text{res}}^{i}$	The residual energy of node *i*
$n_{i,s}^{o}$	The set of *i*'s neighbor nodes that are members of any other cluster in sector *s* ∈ Ψ*_c_*
*n_k,wcs_*	The set of sensor nodes that belong to the working communication sector (wcs) of CH *k*
*W_i,s_*	Cluster head selection weight of sensor *i* in sector *s* ∈ Ψ*_s_*
*G_i,s_*	Gateway selection weight of sensor *i* in sector *s* ∈ Ψ*_s_*
Λ(*i*)	Area covered by any sector *s* ∈ Ψ*_s_* of any node *i* ∈ 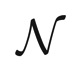
Λ(*i*,*J*)	Overlapping area between nodes *i* and *j*
*O_i_*	Set of nodes having overlapped area coverage with node *i*

**Table 2.** Network configuration parameters.

**Parameters**	**Value**
Simulation Area	1000 m × 1000 m
Deployment Type	Uniform random
Number of Sensor Nodes	200 ∼ 1000
Number of Communication and Sensing Sectors	2∼6
Number of Moving Targets	1∼5
Transmission Range	100 m
Sensing Range	50 m
Target Moving Velocity	1 ∼ 6 m/s
Data Reporting Rate	1 packet/s
Network Bandwidth	512 Kbps
Initial Energy of a Sensor Node	5J
*γ^th^*	1J
*k* (*k*-coverage)	3
Simulation Time	1000 s
